# Characterization of the Polish Primitive Horse (Konik) maternal lines using mitochondrial D-loop sequence variation

**DOI:** 10.7717/peerj.3714

**Published:** 2017-08-24

**Authors:** Jakub Cieslak, Lukasz Wodas, Alicja Borowska, Ernest G. Cothran, Anas M. Khanshour, Mariusz Mackowski

**Affiliations:** 1 Department of Horse Breeding, Poznan University of Life Sciences, Poznan, Poland; 2 Department of Veterinary Integrative Biosciences, College of Veterinary Medicine and Biomedical Science, Texas A&M University, College Station, TX, United States of America; 3 Texas Scottish Rite Hospital for Children, Dallas, TX, United States of America; 4 Horse Genetic Markers Laboratory, Poznan University of Life Sciences, Poznan, Poland

**Keywords:** Polish Primitive Horse, mtDNA D-loop sequence, Maternal line, Genetic diversity, Conservation program

## Abstract

The Polish Primitive Horse (PPH, Konik) is a Polish native horse breed managed through a conservation program mainly due to its characteristic phenotype of a primitive horse. One of the most important goals of PPH breeding strategy is the preservation and equal development of all existing maternal lines. However, until now there was no investigation into the real genetic diversity of 16 recognized PPH dam lines using mtDNA sequence variation. Herein, we describe the phylogenetic relationships between the PPH maternal lines based upon partial mtDNA D-loop sequencing of 173 individuals. Altogether, 19 mtDNA haplotypes were detected in the PPH population. Five haplotypes were putatively novel while the remaining 14 showed the 100% homology with sequences deposited in the GenBank database, represented by both modern and primitive horse breeds. Generally, comparisons found the haplotypes conformed to 10 different recognized mtDNA haplogroups (A, B, E, G, J, M, N, P, Q and R). A multi-breed analysis has indicated the phylogenetic similarity of PPH and other indigenous horse breeds derived from various geographical regions (e.g., Iberian Peninsula, Eastern Europe and Siberia) which may support the hypothesis that within the PPH breed numerous ancestral haplotypes (found all over the world) are still present. Only in the case of five maternal lines (Bona, Dzina I, Geneza, Popielica and Zaza) was the segregation of one specific mtDNA haplotype observed. The 11 remaining lines showed a higher degree of mtDNA haplotype variability (2–5 haplotypes segregating in each line). This study has revealed relatively high maternal genetic diversity in the small, indigenous PPH breed (19 haplotypes, overall HapD = 0.92). However, only some traditionally distinguished maternal lines can be treated as genetically pure. The rest show evidence of numerous mistakes recorded in the official PPH pedigrees. This study has proved the importance of maternal genetic diversity monitoring based upon the application of molecular mtDNA markers and can be useful for proper management of the PPH conservation program in the future.

## Introduction

The Polish Primitive Horse (PPH also known as Konik) is a Polish native horse breed created at the beginning of 20th century on the basis of primitive horses from eastern Poland. The founders of the breed were recruited from the local people who kept the small, tarpan-looking horses mainly because of their low food requirements and good adaptation to the difficult environmental conditions. The start of organized PPH breeding is dated to 1923 when the first individuals were placed in the oldest Polish national horse stud in Janów Podlaski (Jaworski, 1997). Before World War II, the PPH were kept in several breeding centers located in the eastern Poland, including the Białowieża Reserve, established by Professor Tadeusz Vetulani (1936) in order to keep the horses in a presumed natural habitat. Unfortunately, from the population of over 140 PPH individuals which remained in Polish studs at the beginning of German occupation (1939) only about one-third survived the War (Jezierski & Jaworski, 2008). From that time, based predominantly on the above mentioned, decimated PPH population, the breed has been developing strenuously and in 1962 the first official studbook was published (Mackowski et al., 2015). It should be mentioned that in that first studbook, 34 maternal and six paternal lines were included. Over 20 years later an additional line of Geneza mare was delineated. Unfortunately, due to improper supervision of breeding (before the PPH conservation program was established) during almost 40 years of the official studbook existence, 19 of 35 maternal lines were lost and are considered extinct (Jaworski, 1997). Furthermore, the breeding activity of the remaining 16 maternal lines (Białka, Bona, Dzina I, Geneza, Karolka, Liliputka, Misia II, Ponętna, Popielica, Tarpanka I, Traszka, Tunguska, Tygryska, Urszulka, Wola, Zaza) is not equal and some of the 16 are endangered. It should be underlined that since 1985 the PPH studbook is closed and no outside blood is recorded. Since 2000 the Polish Primitive Horse breed is managed within a conservation program, supervised by the National Research Institute of Animal Production (Balice, Poland).

**Figure 1:**
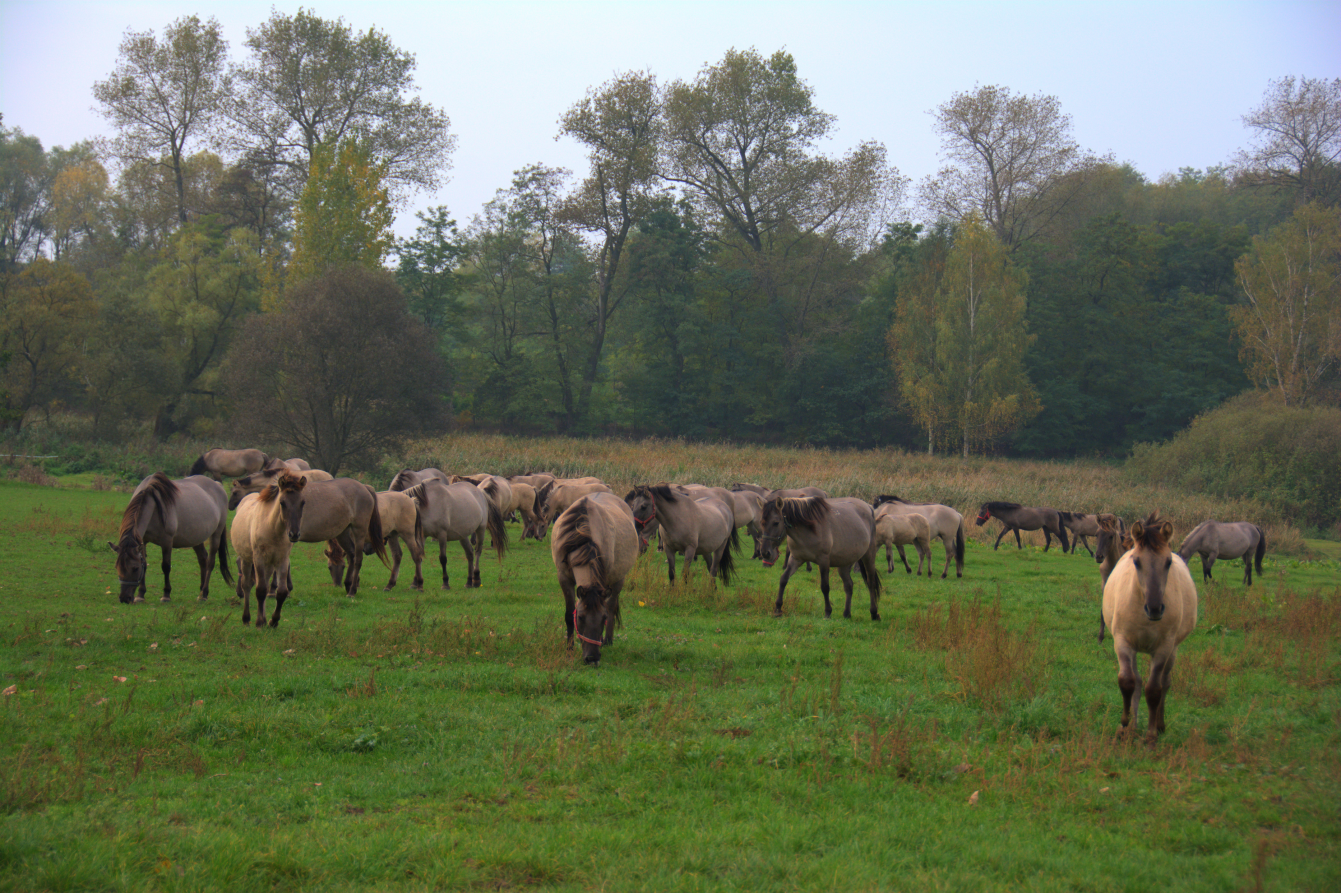
The Polish Primitive Horse herd (Kobylniki national stud, Wielkopolska, Poland. Photo credit: Dr. Grzegorz Cholewinski).

The main features distinguishing PPH from other horse breeds are: limited food requirements, high reproductive parameters and good health (Slivinska, Gawor & Jaworski, 2009). The superior adaptation to the natural habitat, together with the characteristic phenotype of a primitive horse (Fig. 1) (including body conformation, grullo pigmentation etc.) allowed the formulation of the thesis that PPH have putatively inherited a significant part of the wild Tarpan *(Equus caballus gmelini)* traits (Mackowski et al., 2015). Therefore, the PPH breed is considered as a valuable resource of domestic horse biodiversity and for over 15 years has been under a conservation program (Wolc & Balińska, 2010). According to the Polish Horse Breeders Association the current number of the breeding Polish Primitive horses exceeds 1,600 (1,450 broodmares and 160 stallions). These numbers clearly show that for the last two decades the population of Polish Primitive Horse has increased rapidly but if we take into account that the whole breed was derived from a very limited number of founders and the studbook was closed over 30 years ago it is obvious that the genetic diversity parameters of this population should be constantly monitored. This was confirmed by the recent study based upon both pedigree and molecular data, showing an increase of the inbreeding coefficient in PPH during last 30 years. For instance, the mean inbreeding of the PPH breed in 1980 was 4.8% whereas by 2011 it had risen to 8.6% (Mackowski et al., 2015). According to the newest PPH breeding program, to avoid the potential negative effects of inbreeding, the balanced development of all existing maternal and paternal lines is recommended.

There are no previous studies characterizing the genetic diversity in PPH maternal lines based upon mitochondrial DNA sequence variation. Only single PPH individuals were included in previously published investigations regarding multiple horse breeds (Jansen et al., 2002; Cothran, Juras & Macijauskiene, 2005; Cieslak et al., 2010; Georgescu et al., 2011; Kusza et al., 2013). In the recently published work by Szwaczkowski et al. (2016) focused upon the genetic diversity within the PPH breed, the authors claimed that there are no clear genetic differences among the majority of maternal lines. However, this study was based on microsatellite marker analysis which is not the best method for investigation of genetic diversity in dam lines, especially if only a limited number of animals are available. For several reasons (maternal inheritance, lack of recombination, fast molecular evolution and high copy number in the single cell) mitochondrial DNA (mtDNA) analysis is considered the most powerful method recommended for maternal lines phylogenetic studies (Cieslak et al., 2010). Therefore, we decided to perform this study with the main goal to assess the genetic diversity of PPH maternal lines based on the mtDNA D-loop sequence variation. Moreover, based upon obtained results, we assessed the phylogenetic relationship between PPH and other primitive horse breeds.

### Materials and Methods

Altogether 173 DNA samples were used in this study. All samples were derived from the Horse Genetic Markers Laboratory collection (Poznan University of Life Sciences, Poland), where they were previously used for routine parentage control commissioned by horse owners (national and private studs located mainly in Wielkopolska, Silesia, Pomerania, Warmia and Mazury regions). In total, samples originated from PPH mares representing the 16 existing maternal lines. The detailed structure of investigated PPH population is shown in Table 1. Despite that horses representing the same maternal line are related by definition, during the sample selection procedure we tried to avoid close relationship between investigated animals (like mother–daughter or full siblings). It should also be mentioned, that only horses with confirmed parentage (based on microsatellite markers analysis) were included in this study. Affiliation of each individual to a specific maternal line was carefully checked in the official PPH pedigrees, available online in the Polish Horse Breeders Association database (http://baza.pzhk.pl).

**Table 1:** The studied PPH maternal lines and the basic indices describing analyzed mtDNA D-loop sequence variation.

Maternal line	*n*	HapN	HapD (SD)	NPS	π (SD)	*K*
Białka	14	3	0.58 (0.09)	12	0.006 (0.0040)	2.1
Bona	5	1	0	0	0	0
Dzina I	12	1	0	0	0	0
Geneza	11	1	0	0	0	0
Karolka	13	4	0.53 (0.18)	17	0.017 (0.0054)	5.2
Liliputka	14	5	0.73 (0.10)	12	0.010 (0.0027)	3.3
Misia II	7	2	0.29 (0.20)	11	0.009 (0.0065)	3.1
Ponętna	7	3	0.76 (0.12)	13	0.019 (0.0061)	6.3
Popielica	10	1	0	0	0	0
Tarpanka I	13	3	0.60 (0.09)	12	0.014 (0.0023)	4.5
Traszka	11	2	0.18 (0.14)	10	0.005 (0.0043)	1.8
Tunguska	8	2	0.25 (0.18)	10	0.008 (0.0077)	2.5
Tygryska	11	2	0.47 (0.13)	12	0.017 (0.0048)	5.6
Urszulka	13	3	0.30 (0.16)	15	0.010 (0.0051)	3.2
Wola	15	2	0.42 (0.11)	11	0.014 (0.0037)	4.6
Zaza	9	1	0	0	0	0

**Notes.**

HapN number of haplotypes HapD (SD) haplotype diversity with its standard deviation NPS number of polymorphic sites π (SD) nucleotide diversity with its standard deviation *k* average number of nucleotide differences

DNA was extracted from frozen blood samples using MasterPure™ Complete DNA and RNA Purification Kit (Epicentre, Madison, WI, USA), according to the manufacturer’s protocol. The quantity and quality of the obtained DNA was tested using NanoDrop 2000 spectrophotometer (Thermo Scientific, Waltham, MA, USA).

PCR was conducted using primers described before (Cothran, Juras & Macijauskiene, 2005). Amplification of 510 bp fragment of the equine mitochondrial DNA sequence (nucleotide positions: 15343–15852, according to GenBank X79547 reference sequence), containing the HVR1 (hypervariable region 1) was carried out in 10 µl total volume containing: 1U of DreamTaq DNA Polymerase (Thermo Fisher Scientific, Waltham, MA, USA), 0.5 mM of each primer, 1 × PCR buffer, 0.2 mM of dNTPs and 50 ng of template DNA. PCR was conducted in T-100 Thermal Cycler (BioRad, Hercules, CA, USA) using the following conditions: initial denaturation (95 °C, 5 min); 25 cycles of denaturation (95 °C, 45 s), primers annealing (58 °C, 45 s), elongation (72  °C, 45 s); final elongation (72 °C, 10 min). The quantity and integrity of PCR products were examined in 1.5% agarose gel stained with ethidium bromide (120 V, 25 min). Afterwards, PCR products were cleaned from unused primers and nucleotides by FastAP Thermosensitive Alkaline Phosphatase and Exonuclease I (Thermo Fisher Scientific, Waltham, MA, USA) digestion (37 °C, 30 min; 80 °C, 15 min). The sequencing reaction (based on the BigDye® Terminator v1.1 Cycle Sequencing Kit; Thermo Fisher Scientific, Waltham, MA, USA) was performed in the above-mentioned thermocycler in the following conditions: initial denaturation (95 °C, 5 min); 25 cycles: of denaturation (95 °C, 30 s), primer annealing (50 °C, 10 s) and elongation (60  °C, 4 min). The sequencing reaction was based on the same primer (forward) as used for PCR amplification. For samples giving not fully readable results and each rare haplotype the sequencing was carried out in both directions. In the next step samples were filtered through 96-well plate with Sephadex (Sigma Aldrich, Germany) by centrifugation (3,180× g, 3 min) and separated in ABI Prism 3130 Genetic Analyzer (Applied Biosystems, Foster City, CA, USA). Finally, obtained electropherograms were analyzed in Lasergene SeqMan Pro (version 12.2.0) software (DNASTAR, Madison, WI, USA).

For PPH phylogenetic analysis purposes the 333 bp fragment including all detected polymorphic sites was used. Obtained sequences were aligned with the application of MegAlign Pro software (DNASTAR, Madison, WI, USA) using the GenBank X79547 mtDNA sequence as reference. The basic indices describing analyzed D-loop sequence polymorphism (number of polymorphic sites, number of haplotypes, haplotype diversity, nucleotide diversity and average number of nucleotide differences) were calculated with the application of the DnaSP (version 5.10.1) tool (Librado & Rozas, 2009). Haplotypes segregating in the investigated PPH population were compared with other equine mtDNA sequences deposited in the GenBank database using BLAST. Separately, in order to assign the described haplotypes to the specific haplogroups the comparison with sequences derived from the study by Achilli et al. (2012) was carried out. Finally, to show the phylogenetic context of the PPH breed comparison with 79 mtDNA sequences (deposited in GenBank) derived from 14 different European indigenous horse breeds as well as the Yakutian horse (primitive horse breed from the Siberia region) was carried out. Only sequences covering fully the 333 bp D-loop fragment analyzed in the present experiment were taken into account. On this basis the consensus phylogenetic tree of the D-loop haplotypes was constructed using PHYLIP package (Felsenstein, 1993) with the application of Neighbor-joining method (Saitou & Nei, 1987) with 1,000 bootstrap replications. In order to fully reflect the phylogenetic relations between all 173 tested D-loop sequences, another neighbor-joining tree (without bootstrapping) was drawn. Moreover, to describe the phylogenetic relationships between the analyzed PPH haplotypes and D-loop sequences of other European primitive horse breeds, the median-joining (MJ) network was constructed in the NETWORK 5 software (http://www.fluxus-engineering.com) using the default settings. The mutational hot spots present in the analyzed D-loop fragment were taken into consideration and down-weighted into 0.5, as done in previously published studies (Jansen et al., 2002; Cieslak et al., 2010).

## Results

Altogether 33 variable sites (32 substitutions and one InDel polymorphism) were detected in the analyzed mtDNA D-loop sequence fragment. These segregated in the PPH population in the form of 19 haplotypes (Table 2). The studied haplotypes are numbered as H_1–H_20, where H_1 corresponds to the reference mtDNA sequence (GenBank X79547).

Comparison of the results with mtDNA sequences deposited in the GenBank database using BLAST showed that all detected polymorphic sites were previously described by other authors. Interestingly, the detailed analysis proved that A>G substitution at position 15672 segregates exclusively in PPH breed. The two other polymorphic sites (15726A>G, 15807T>C) are characteristic for PPH and single sequences derived from indigenous horse breeds from Asia (e.g., China and Mongolia). Separate analysis of 19 PPH haplotypes indicated that 5 of them (H_2, H_8, H_13, H_14 and H_16) are potentially novel whereas the remaining 14 show 100% homology with sequences previously deposited in GenBank derived from both modern (e.g., Irish drought horse, Thoroughbred, Lipizzan) and primitive (e.g., Hucul, Jeju, Yakutian) horse breeds. It should be stressed that the new haplotypes differ from others mainly due to the presence of the above mentioned rare polymorphisms (15672A>G, 15726A>G and 15807T>C).

The haplotype diversity (HapD) calculated for all investigated horses was 0.92, while the nucleotide diversity (π) and the average number of nucleotide differences (*k*) were 0.026 and 8.4, respectively. Similar calculations conducted separately for each maternal line showed that only in the case of 5 PPH dam lines (Bona, Dzina I, Geneza, Popielica and Zaza) a single, characteristic mtDNA haplotype can be assigned (Table 1). Among the remaining 11 lines the segregation of 2–5 haplotypes was observed, with the HapD index varying within the range of 0.18 (Traszka) and 0.76 (Ponętna). An identical trend was observed for the *k* and π parameters with the lowest values (*k* = 1.8; π=0.005) noticed for Traszka line and the highest indices (*k* = 6.3; π = 0.019) calculated for individuals representing Ponętna line. It should also be noted that the proportion of horses carrying different haplotypes varied among the investigated maternal lines. For example, in the Urszulka line (*n* = 13) about 85% of horses had haplotype H_13 and only two individuals had other haplotypes (H_4 and H_12) assigned. On the other hand, in the Ponętna line (*n* = 7) the observed frequencies of the three segregating haplotypes were more balanced (H_15—42%, H_4—29% and H_13—29%). Interestingly, in the Liliputka and Karolka lines we found single horses carrying 4 unique haplotypes (H_14, H_18–20) which were not present in any other studied animals. Moreover, in the Białka maternal line the two haplotypes (H_2 and H_16) were observed, and these were found exclusively in this group. The detailed distribution of the detected haplotypes in all investigated PPH dam lines is shown in Table S1.

**Table 2:** Detected polymorphic sites and PPH haplotypes. Mutational hotspots (according to Cieslak et al., 2010) are marked in red.

Haplotype (GenBank acc. number)	Nucleotide position (based on GenBank X79547 reference sequence)																																
	15532	15542	15585	15597	15598	15601	15602	15604	15615	15616	15617	15635	15650	15659	15666	15667	15672	15703	15720	15726	15740	15770	15771	15775	15776	15777	15806	15807	15809	15810	15811	15826	15827
1 (X79547 –ref.)	C	C	G	A	T	T	C	G	A	A	T	C	A	T	G	A	G	T	G	G	A	C	C	C	T	A	C	C	A	A	C	A	A
2 (MF120550)	.	T	.	G	.	.	T	.	.	.	.	.	G	.	A	.	.	.	A	A	.	.	T	.	.	.	.	.	.	G	.	.	.
3 (MF120551)	.	.	A	.	.	.	T	A	.	.	.	.	.	.	.	.	.	C	A	.	G	.	T	.	.	G	.	.	.	.	T	.	.
4 (MF120530)	.	.	.	.	.	.	.	.	.	.	.	.	.	.	.	.	.	.	.	.	.	.	.	.	.	.	.	.	.	.	.	G	.
5 (MF120531)	.	.	A	.	.	.	.	.	.	.	.	.	G	.	A	.	.	.	A	.	.	.	.	.	.	.	.	.	.	G	.	G	.
6 (MF120532)	.	T	.	G	.	.	T	.	.	.	.	.	G	.	A	.	.	.	A	.	.	.	T	.	.	.	.	.	.	.	.	.	.
7 (MF120533)	.	.	A	.	.	C	T	.	.	.	.	.	.	.	.	.	.	.	A	.	.	.	T	.	.	.	T	.	.	.	.	.	G
8 (MF120534)	.	.	.	G	.	.	T	A	.	.	.	.	.	.	.	G	.	C	A	.	.	.	T	.	.	G	.	T	G	.	.	.	.
9 (MF120535)	.	.	.	.	.	.	.	.	.	.	C	.	G	.	A	.	.	.	A	.	.	.	.	.	.	.	.	.	.	.	.	G	.
10 (MF120536)	.	.	.	.	.	.	T	.	.	.	C	.	.	C	.	.	.	.	A	.	.	.	T	.	.	.	T	.	.	.	.	.	G
11 (MF120537)	.	.	.	.	C	.	T	.	G	G	.	.	.	.	.	.	.	C	A	.	.	T	.	T	.	.	T	.	.	.	.	.	G
12 (MF120538)	DEL	.	A	.	.	.	T	.	.	.	.	.	.	.	.	.	.	.	A	.	.	.	T	.	.	.	.	T	.	.	.	.	G
13 (MF120539)	.	T	.	G	.	.	T	.	.	.	.	T	G	.	A	.	A	C	A	.	.	.	.	.	.	.	.	.	.	.	.	.	.
14 (MF120540)	.	.	A	.	.	C	T	.	.	.	.	.	.	.	.	.	.	.	A	A	.	.	T	.	.	.	T	.	.	.	.	.	G
15 (MF120541)	.	.	A	.	.	.	T	A	.	.	.	.	.	.	.	.	.	C	A	A	G	.	T	.	.	G	.	.	.	.	T	.	.
16 (MF120542)	.	T	.	G	.	.	T	.	.	.	.	.	G	.	A	.	.	.	A	A	.	.	T	.	.	.	.	.	.	.	.	.	.
17 (MF120543)	.	.	.	.	C	.	T	.	G	G	.	.	.	C	.	.	.	C	A	.	.	T	.	T	C	.	T	.	.	.	.	.	G
18 (MF120544)	.	.	.	.	.	.	.	.	.	.	.	.	G	.	A	.	.	.	A	.	.	.	.	.	.	.	.	.	.	.	.	G	.
19 (MF120545)	.	T	A	G	.	.	T	.	G	.	.	T	G	.	A	.	.	C	A	.	.	.	.	.	.	.	.	.	.	.	.	.	.
20 (MF120546)	.	.	.	.	.	C	T	.	.	.	.	.	.	.	.	.	.	.	A	.	.	.	T	.	.	.	T	.	.	.	.	.	G

**Figure 2:**
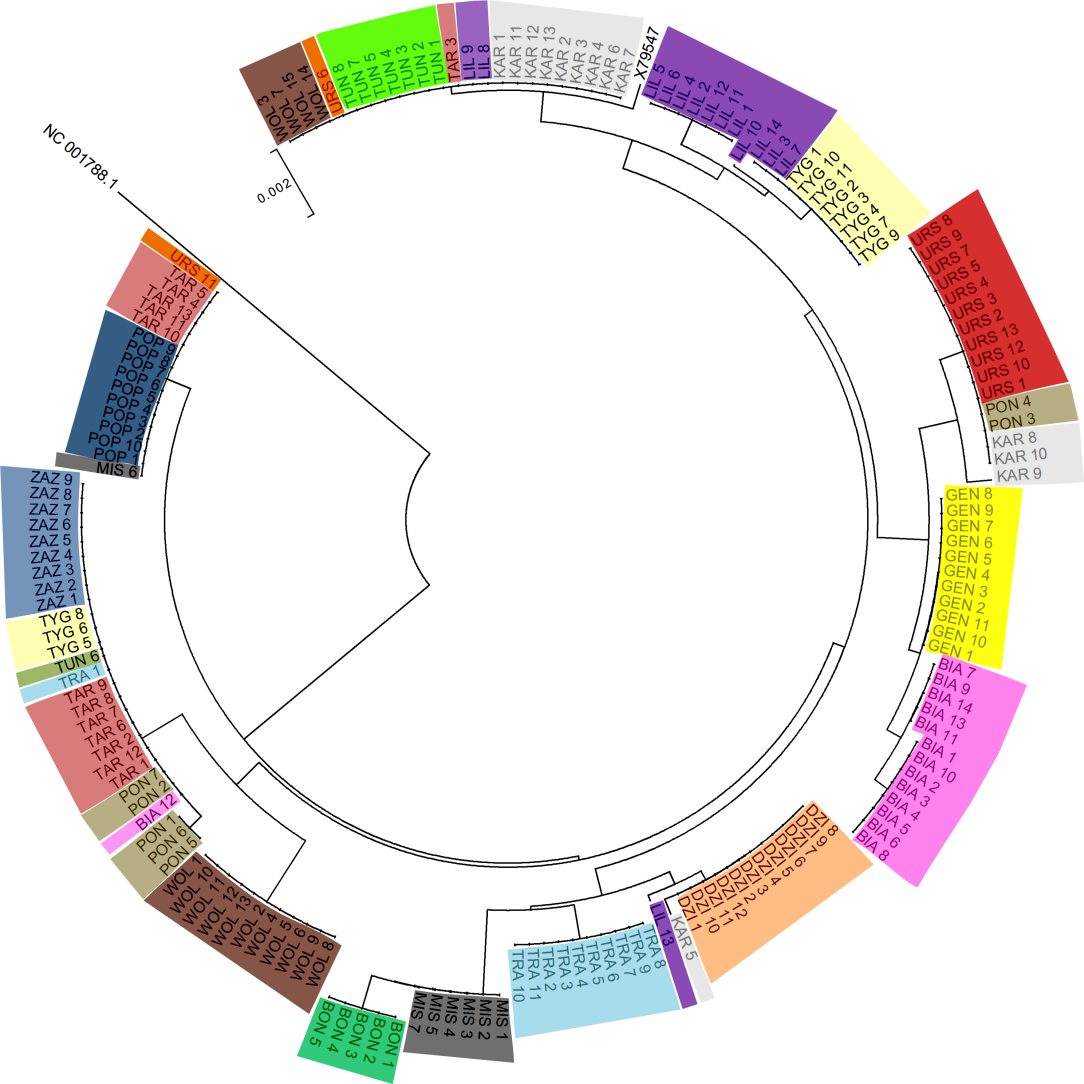
The Neighbor-joining tree of the 175 tested mtDNA D-loop sequences. Individuals sharing the maternal line (according to the pedigree data) are marked with the same color and dam line acronym.

Figure 2 shows the Neighbor-joining tree of 175 samples (173 PPH individuals, the reference X79547 equine mtDNA sequence, and the donkey NC 001788.1 mtDNA sequence as an out-group). The tree clearly reflects the grouping of sequences into multiple clusters represented by different numbers of horses. The simple comparison of the observed clusters sizes with the number of animals classified previously to the separate maternal lines (Table 1), suggests that obtained results do not correspond fully to the official PPH pedigree data. For example, the maximal number of horses assigned initially to one maternal line was 15 (Wola) whereas on the tree we can find two clusters exceeding 20 individuals which share the identical D-loop sequence. This indicates that in several cases the same mtDNA haplotype is shared by horses representing different maternal lines (according to the pedigree data).

**Figure 3:**
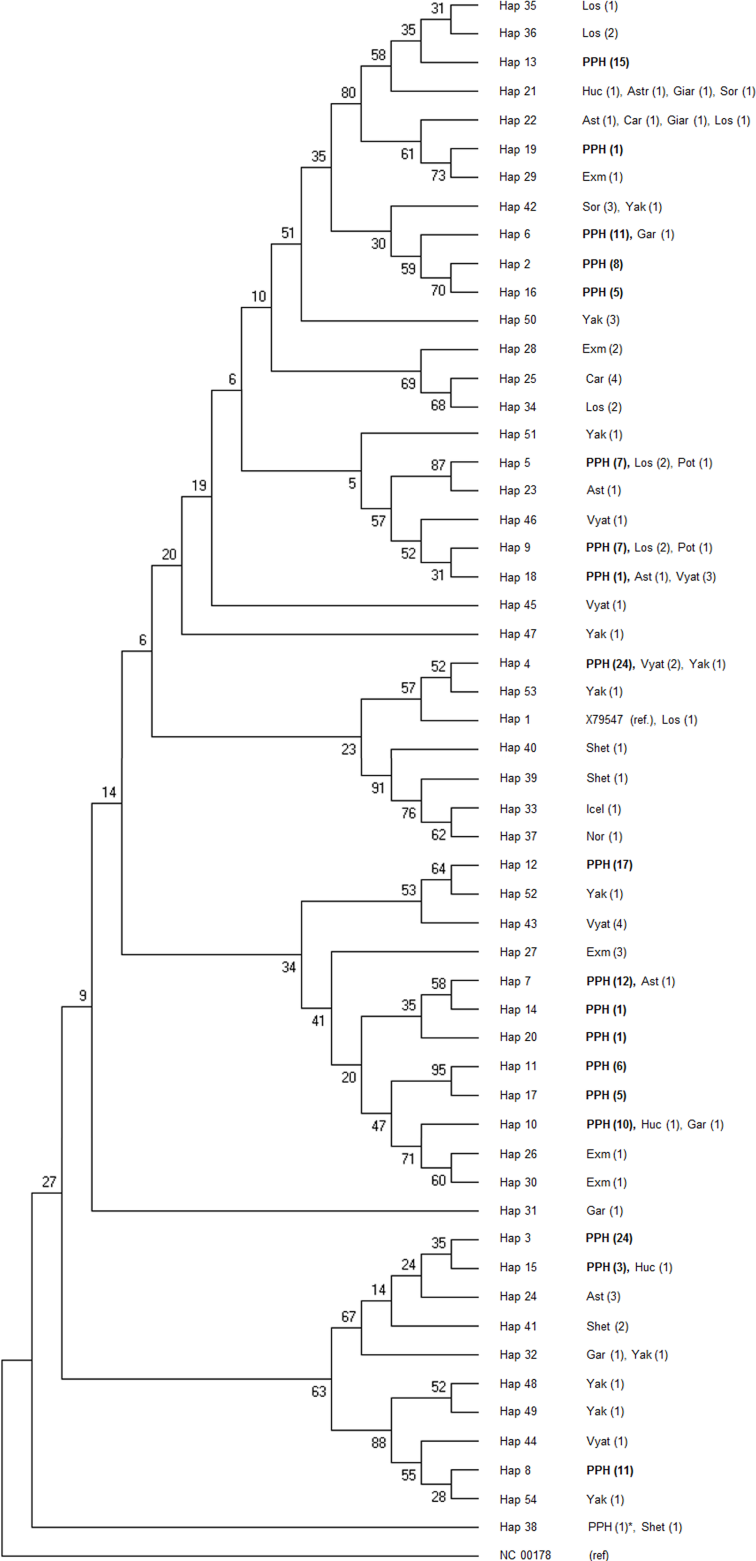
Consensus Neighbor-joining tree of the 19 haplotypes found in PPH maternal lines and sequences of other primitive horse breeds. The donkey reference sequence was used as the out-group. At each branch of the tree the breeds acronyms are shown together with the number of studied individuals (in brackets). The PPH acronym at haplotypes 2–20 (described in this study) is shown in bold. The only PPH sequence derived from the GenBank database is marked with asterisk. The tree was constructed based upon 1,000 bootstrap replications. Bootstrap values are shown as percentages. Horse breeds acronyms: Ast, Asturcon; Car, Cartujano; Exm, Exmoor; Gar, Garrano; Giar, Giara; Icel, Icelandic Horse; Los, Losino; Nor, Norwegian Fjord; Shet, Shetland Pony; Sor, Sorraia; Vyat, Vyatskaya; Yak, Yakutian; PPH, Polish Primitive Horse; Huc, Hucul Horse; Pot, Pottoka.

**Figure 4:**
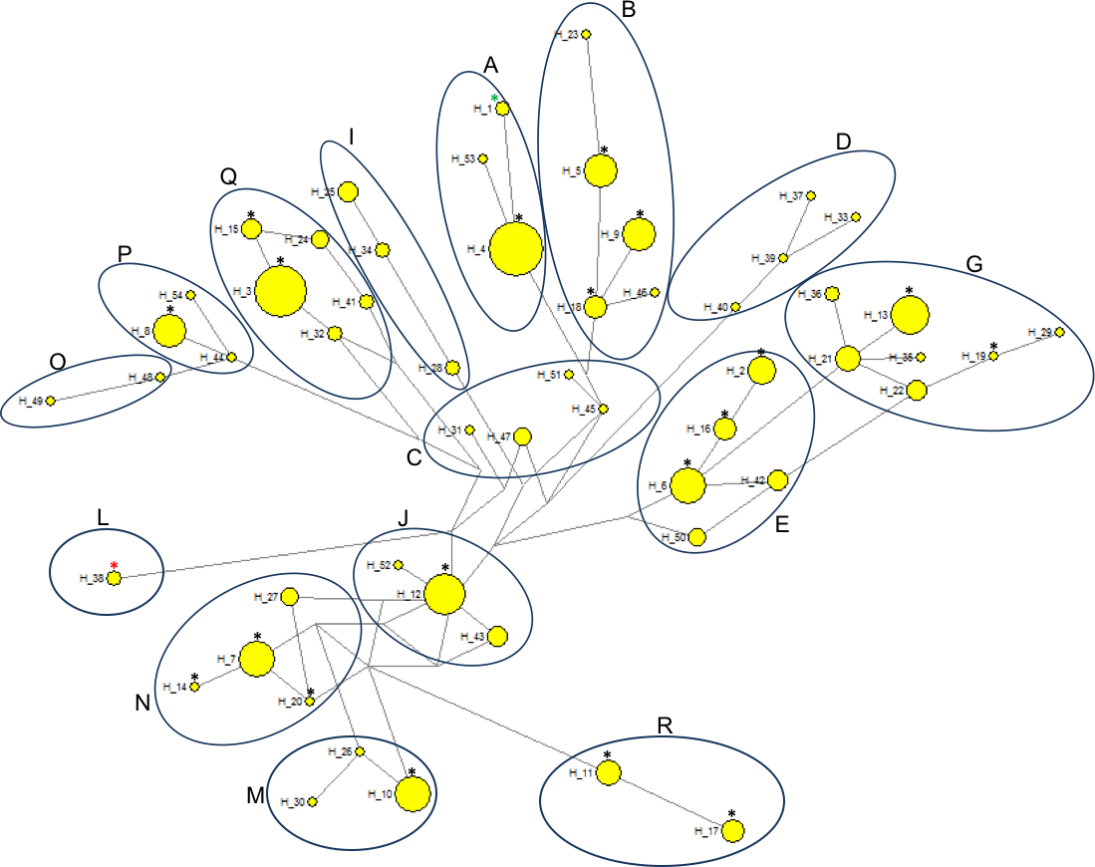
Median-joining network based on 333 bp of the mitochondrial D-loop sequence representing 173 horses within 20 haplotypes. The haplogroups were named according to Achilli et al. (2012). The sizes of the circles are proportional to the number of individuals carrying given haplotype. Black asterisks, PPH haplotypes tested in the present study (H_2—H_20); red asterisk, haplotype of the only PPH sequence derived from the GenBank database (HQ439473); green asterisk, reference equine mtDNA sequence (X79547).

Phylogenetic relationships between 19 PPH haplotypes tested in this study and 79 D-loop sequences (GenBank) derived from 15 indigenous horse breeds were illustrated by the Neighbor-joining tree (Fig. 3). Bootstrap values were generally not high (range: 5–95%), but if compared to the shape of the median-joining network tree (Fig. 4) a high level of similarity in haplotype groupings is present. This indicates that the predicted model of phylogeny is putatively correct. As was shown on Fig. 3, PPH share identical mtDNA haplotypes with primitive horses from various geographical regions including Iberian Peninsula (Asturcon, Garrano, Sorraia, Losino, Cartujano, Pottoka), Sardinia (Giara), Eastern Europe (Hucul, Vyatskaya) and Siberia (Yakutian). Interestingly, the haplotype H_38 derived from the only analyzed PPH sequence (HQ439473) from GenBank database does not correspond to any of 19 haplotypes described in this work but is identical with one of the Shetland Pony D-loop sequences deposited in GenBank (AF481295) derived from paper by Hill et al. (2002). It may indicate that the HQ439473 studied in the experiment by Lippold et al. (2011) is derived from a not genetically pure PPH individual. This hypothesis is partly supported by results shown on Fig. 4 because only this sequence (and above mentioned identical Shetland Pony D-loop fragment) were assigned to the haplogroup L. None of the remaining 251 mtDNA sequences used in this study (including 173 PPH individuals) were allocated to this particular haplogroup. On the other hand, it cannot be excluded that the above mentioned sequence is derived from one of the PPH maternal lines which is now considered extinct.

Comparative analysis of the results obtained in the present study with previously published horse mtDNA data has shown that haplotypes found in PPH maternal lines can be assigned to 10 (A, B, E, G, J, M, N, P, Q and R) out of 18 major haplogroups described by Achilli et al. (2012) (Fig. 4). Interestingly, in our PPH breed sample set derived from 173 horses we did not find haplotypes belonging to haplogroup L, which is known to be the most frequent in Europe and the Middle East. On the other hand, H_12 was assigned to the haplogroup J, which is very seldom seen in the European horse population (Achilli et al., 2012). A closer look at the affiliation of sequences derived from horses representing separate PPH maternal lines to the various haplogroups confirms the hypothesis about numerous mistakes present in PPH pedigrees. For example, within the Białka line we have found individuals carrying H_8 and H_16, whereas these haplotypes represent phylogenetically distinct haplogroups (P and E, respectively).

## Discussion

The unquestionable utility of mtDNA sequence analyses in phylogenetic studies of different species is well known. In the case of the domestic horse, the retrospective analyses of material derived from fossils and their comparison with modern horse breeds mtDNA data, have precisely depicted the history of mitochondrial DNA lineages before and after domestication (Cieslak et al., 2010; Achilli et al., 2012). As was indicated by Cieslak et al. (2010), the relatively high level of mitochondrial DNA diversity in the domestic horse (numerous mtDNA haplotypes segregating in the modern horse breeds) is probably due to multiple incidents of domestication and/or introgression of mares. The number of ancient haplotypes increased especially during the Iron Age, which could be an effect of a second wave of domestication. These results are generally in concordance with previously published findings by Jansen et al. (2002) who concluded that at least 77 mares were recruited from the wild and suggested that probably several distinct horse populations were involved in the domestication process.

Besides the above mentioned experiments showing the natural history of the whole domestic horse species, mtDNA sequencing is an important tool in studies regarding the maternal genetic diversity in local, endangered horse breeds (Cothran, Juras & Macijauskiene, 2005; Moridi et al., 2012; Bigi, Perrotta & Zambonelli, 2014; Sziszkosz et al., 2016). To our knowledge, there were no previous studies focused on the mtDNA sequence variation in all 16 Polish Primitive Horse maternal lines. Because in the PPH conservation program the emphasis is placed on preservation and an even genetic contribution of founder active dam lines (http://www.bioroznorodnosc.izoo.krakow.pl), monitoring of the real maternal diversity with the application of mtDNA markers seems to be necessary. As was shown before (Mackowski et al., 2015) the level of inbreeding in PPH population is increasing, which is related mainly to the limited number of PPH founders. The genetic pool of the breed may also be partly diminished because of long-term selection for grullo (blue dun) coat color, since the animals with different pigmentation types (including horses with white markings) cannot, by rule, be recorded in the official PPH studbook. In addition, despite the fact that one of the main goals of the PPH conservation program is to preserve the characteristic primitive phenotype of the breed, the recently published report by Pasicka et al. (2017) has proved that during almost 100 years of managed PPH breeding some of the body conformation features have changed significantly. This is probably related to more or less intentional selection for saddle utility related traits.

Taking into account the results obtained in the present study it is clear that the overall maternal genetic diversity in the whole PPH breed is relatively high since 19 different haplotypes were detected. The calculated haplotype diversity (0.92) is similar to many previously published reports regarding various horse breeds: Arabian—HapD = 0.97 (Khanshour & Cothran, 2013), Hungarian Gidran Horse—HapD = 0.91 (Sziszkosz et al., 2016), Hucul—HapD = 0.94 (Kusza et al., 2013) and is higher than observed in some of European and Asiatic native breeds, like: Monterufolino—HapD = 0.80 (Cardinali et al., 2016) or Kiso Horse—HapD = 0.79 (Takasu et al., 2014). Also the nucleotide diversity parameter estimated in the present study (π = 0.026) corresponds with other studies regarding multiple modern horse breeds (Achilli et al., 2012) and is within the range (0.005–0.034) presented for ancient horse populations by Cieslak et al. (2010). As well, previously published reports based upon a single PPH individual likely do not accurately reflect the phylogenetic relationship of the PPH to other horse breeds. Georgescu et al. (2011) have shown that on the phylogenetic tree the PPH is located in the main cluster, together with many other breeds (Icelandic Pony, Exmoor, Shetland Pony, Akhal Teke and Caspian Pony) and separately from the cluster including Hucul horses (the other primitive European horse breed). However, in the article by Kusza et al. (2013) the PPH individuals, as well as the Hucul horses, were dispersed in many different clusters of the tree, and dependent on the carried haplotype, have shown higher similarity to other primitive or modern horse breeds. Since the above mentioned investigations were based on very limited number of PPH-derived samples (4–7 individuals) we decided to compare our sample set with GenBank sequences of multiple local (mainly European) horse breeds. On the basis of previously published reports we can conclude that the distribution of the particular haplogroups is uneven among different geographical regions of the world. 5 of 19 tested PPH haplotypes (H_3, H_4, H_13, H_15 and H_19) were assigned to the haplogroups A, G and Q which according to literature data occur most frequently in Asia (Achilli et al., 2012). On the other hand, we expected the high frequency of D-loop haplotypes belonging to the haplogroup L (considered the most prevalent in Europe) while in fact, within our broad PPH sample set, we did not find sequences which would be classified to this group. These findings will provide new insight on the understanding of PPH breed phylogenetic origin, which is still considered unclear. A closer look at the results of interbreed comparisons of the 19 PPH haplotypes indicates that in majority of cases the identical or similar D-loop sequences are shared by numerous modern and indigenous horse breeds representing different (sometimes very distinct) geographical regions. This may support the speculation that within the small PPH breed a significant part of ancestral equine mtDNA haplotypes (currently dispersed all over the world) is still present.

Despite that there are seven horse breeds covered by conservation programs in Poland, only two (Polish Primitive Horse and Hucul) can be considered as genetically unique and pure, because their studbooks were closed for many years and no other blood is accepted. Three of the other breeds are crossbred with Thoroughbreds at some point in their history and the other two are heavy horse breeds with close relationship to other draft horse breeds. Therefore, the well-defined breeding strategies and continuous monitoring of the genetic diversity should be an important tool helping to maintain the genetic resources of both breeds (Mackowski et al., 2015). However, in the case of PPH the realization of the conservation program assumptions is difficult because the population is relatively small and animals are distributed in multiple places in Poland (Szwaczkowski et al., 2016). Moreover, it is hard to combine the main aims of the program—to keep the overall genetic diversity of the breed on a safe level and to preserve the high level of differentiation between existing maternal lines. Herein we have shown that for five PPH maternal lines (Bona, Dzina I, Geneza, Popielica and Zaza) the assignment of unique mtDNA haplotype is possible. There are also several maternal lines in which the highly uneven distribution of haplotypes allows prediction of the most probable haplotype characteristic for the given line (Liliputka, Misia II, Traszka, Tygryska, Urszulka and Wola). These results are not consistent with the majority of conclusions presented in the recent study by Szwaczkowski et al. (2016). In the cited article regarding the inter- and intra-genetic diversity in the PPH breed the authors did not find significant genetic differences among the majority of maternal lines and therefore they have partly undermined the understanding of the value of the PPH dam lines in the conservation program. Also, the use of microsatellite markers on a very limited group of animals will not accurately estimate the real maternal genetic diversity. For example, in the study by Szwaczkowski et al. (2016) individuals representing the Misia II maternal line were not clustered together on the phylogenetic tree, whereas in our study the above mentioned line presented a relatively high level of divergence with over 85% individuals carrying haplotype 11 (which was not present in any other investigated line). Such discrepancies may be an effect of mating mares representing a given maternal line with different stallions, which is obviously reflected in microsatellite markers genotypes but not in the mtDNA sequence derived data. Despite that there are many differences between these two investigations, their major conclusion is similar—the PPH conservation program needs an extensive revision, mainly related to maternal lines preservation. As was shown in the present study, the majority of PPH maternal lines are more or less “contaminated” by individuals derived from the other line(s). This is obviously an effect of numerous mistakes implemented to the official PPH pedigrees, putatively before the era of the common parentage control. Such errors, reported also for other horse breeds such as Arabian horses, Thoroughbred and Hucul (Khanshour & Cothran, 2013; Hill et al., 2002; Kusza et al., 2013), may have a great impact on the effective maintenance of genetic diversity. Thus, in many cases it should be mandatory to incorporate the results of this very simple and robust technique of mtDNA sequence analysis to the breeding practice. On the other hand, in the case of PPH, the inconsistencies found in the official pedigrees may paradoxically support the hypothesis that one or more maternal line which are commonly considered extinct are in fact still active in the breeding population but are “hidden” because of the pedigree mistakes made several generations before. This is supported by the fact that in our study we have found more mtDNA haplotypes than expected if the number of formally existing PPH maternal lines was taken into consideration (19 *vs.* 16). It should be stressed that a similar find of the lost Sorraia dam line in the Lusitano horse breed was described previously (Luís et al., 2006). Therefore, it would be interesting to perform a similar study in the future, but on a larger animal set and with the use of longer mtDNA sequences.

## Conclusions

Obtained results revealed the presence of a relatively high degree of overall maternal genetic diversity among PPH breed. The analyses based on mtDNA D-loop sequence variation confirmed once again the utility of such an approach in the phylogenetic studies of maternal lines in the domestic horse. The detected significant discrepancies between pedigree and molecular data are convincing evidence that the PPH conservation program, which is concentrated mainly on the protection and development of the 16 existing maternal lines, needs an extensive revision (even the re-division of the PPH maternal lines using the mtDNA derived data should be considered). Otherwise, the distinguishing of PPH dam lines will continue based upon the breeders tradition only, not supported by the valuable pedigree data.

##  Supplemental Information

10.7717/peerj.3714/supp-1 Supplemental Information 1 Distribution of the detected haplotypes among 16 PPH maternal lines (number of individuals)

10.7717/peerj.3714/supp-2 Data S1 Raw data (analyzed FASTA sequences)

## Additional Information and Declarations

### Data Availability

The following information was supplied regarding data availability: The raw data has been provided as a Supplemental File.
