# Supplementary material for: Characterization of the Polish Primitive Horse (Konik) maternal lines using mitochondrial D-loop sequence variation
Source: PeerJ. 2017 Aug 24;5:e3714. doi: 10.7717/peerj.3714 (PMC5572418; doi:10.7717/peerj.3714)
Supplement: Supplemental Information 1 [file peerj-05-3714-s001.pdf]

Supplemental material 1. Distribution of the detected haplotypes among 16 PPH maternal lines (number of individuals)

Haplotype	PPH maternal line															
	Bia (n=14)	Bon (n=5)	Dzi (n=12)	Gen (n=11)	Kar (n=13)	Lil (n=14)	Mis (n=7)	Pon (n=7)	Pop (n=10)	Tar (n=13)	Tra (n=11)	Tun (n=8)	Tyg (n=11)	Urs (n=13)	Wol (n=15)	Zaz (n=9)
**H_1 X79547 ref. (n=0)**	0	0	0	0	0	0	0	0	0	0	0	0	0	0	0	0
**H_2 (n=8)**	8	0	0	0	0	0	0	0	0	0	0	0	0	0	0	0
**H_3 (n=24)**	1	0	0	0	0	0	0	2	0	7	1	1	3	0	0	9
**H_4 (n=24)**	0	0	0	0	9	2	0	0	0	1	0	7	0	1	4	0
**H_5 (n=7)**	0	0	0	0	0	7	0	0	0	0	0	0	0	0	0	0
**H_6 (n=11)**	0	0	0	11	0	0	0	0	0	0	0	0	0	0	0	0
**H_7 (12)**	0	0	12	0	0	0	0	0	0	0	0	0	0	0	0	0
**H_8 (n=11)**	0	0	0	0	0	0	0	0	0	0	0	0	0	0	11	0
**H_9 (n=11)**	0	0	0	0	0	3	0	0	0	0	0	0	8	0	0	0
**H_10 (n=10)**	0	0	0	0	0	0	0	0	0	0	10	0	0	0	0	0
**H_11 (n=6)**	0	0	0	0	0	0	6	0	0	0	0	0	0	0	0	0
**H_12 (n=17)**	0	0	0	0	0	0	1	0	10	5	0	0	0	1	0	0
**H_13 (n=15)**	0	0	0	0	2	0	0	2	0	0	0	0	0	11	0	0
**H_14 (n=1)**	0	0	0	0	1	0	0	0	0	0	0	0	0	0	0	0
**H_15 (n=3)**	0	0	0	0	0	0	0	3	0	0	0	0	0	0	0	0
**H_16 (n=5)**	5	0	0	0	0	0	0	0	0	0	0	0	0	0	0	0
**H_17 (n=5)**	0	5	0	0	0	0	0	0	0	0	0	0	0	0	0	0
**H_18 (n=1)**	0	0	0	0	0	1	0	0	0	0	0	0	0	0	0	0
**H_19 (n=1)**	0	0	0	0	1	0	0	0	0	0	0	0	0	0	0	0
**H_20 (n=1)**	0	0	0	0	0	1	0	0	0	0	0	0	0	0	0	0

Maternal lines acronyms: Bia – Białka, Bon – Bona, Dzi – Dzina I, Gen – Geneza, Kar – Karolka, Lil – Liliputka, Mis – Misia II, Pon – Ponętna, Pop – Popielica, Tar – Tarpanka I, Tra – Traszka, Tun – Tunguska, Tyg – Tygryska, Urs – Urszulka, Wol – Wola, Zaz – Zaza
